# Narrow QRS Tachycardia: What Is the Mechanism?

**DOI:** 10.19102/icrm.2021.120803

**Published:** 2021-08-15

**Authors:** Krishna Kumar Mohanan Nair, Narayanan Namboodiri, Ajitkumar Valaparambil

**Affiliations:** ^1^Department of Cardiology, Sree Chitra Tirunal Institute for Medical Sciences and Technology, Thiruvananthapuram, Kerala, India

**Keywords:** Atrioventricular reentrant tachycardia, narrow QRS tachycardia, premature ventricular beat

## Abstract

A 45-year-old man was referred for radiofrequency catheter ablation of narrow QRS tachycardia that terminated with intravenous adenosine. A 12-lead electrocardiogram showed no baseline pre-excitation. The echocardiogram was essentially normal. The electrophysiological study showed a normal atrial–His interval of 110 ms and a His–ventricular interval of 44 ms during sinus rhythm. An anterograde study demonstrated no dual atrioventricular nodal physiology. Atrial pacing protocols easily and reproducibly induced a narrow QRS tachycardia. What is the mechanism of the tachycardia?

The tachycardia represents a regular narrow QRS tachycardia with discernible P-waves on the ST segment away from the QRS **(Figures 1 and 2)**. The likely differentials for this narrow QRS tachycardia from the surface electrocardiogram were atypical atrioventricular (AV) nodal reentrant tachycardia (AVNRT), orthodromic AV reentrant tachycardia (AVRT) involving a right-sided accessory pathway, and right atrial tachycardia (AT).

This was followed by a spontaneous premature ventricular beat (PVB) from the right ventricular apex with a coupling interval of 315 ms **(Figure 2)**. The premature beat advanced the following “P”-wave (as evident in lead III, **Figure 2**) and reset the tachycardia. The PVB was late-coupled and would be most unlikely to reset AT. Not only that, a short pre-excitation index of 43 ms is consistent with AV reentry. This observation makes AV reentry the most probable mechanism of the narrow QRS tachycardia.^1^

The aforementioned observations from the surface electrocardiogram were confirmed from the intracardiac tracings **(Figure 3)**. The intracardiac recordings showed a 1:1 A–V relation, stable tachycardia cycle lengths, and ventriculo-atrial intervals. The atrial activation pattern was concentric. In response to the spontaneous and late PVB, there was an advancement of the atrial electrogram with an identical atrial activation pattern and a pre-excitation index of 40 ms. A pre-excitation index below 45 ms virtually rules out AVNRT and left-sided AVRT.^2^ Subsequent resetting of the tachycardia on the next tachycardia beat was concealed, probably due to decremental antegrade nodal conduction. Hence, the narrow QRS tachycardia represents an orthodromic AVRT involving a concealed right-sided accessory pathway, which was mapped at the 12 o’clock position of the tricuspid annulus and successfully ablated.

## Figures and Tables

**Figure 1:**
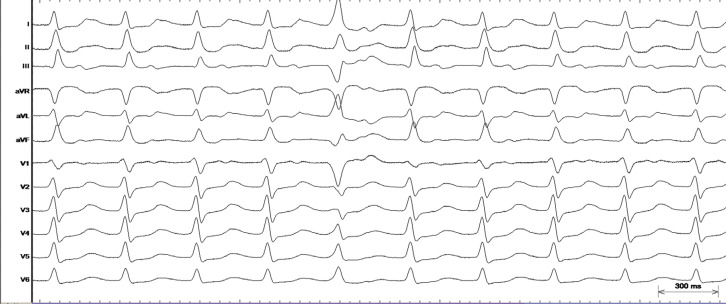
Surface electrocardiogram (I, II, III, aVR, aVL, aVF, V1, V2, V3, V4, V5, V6) during the narrow QRS tachycardia.

**Figure 2:**
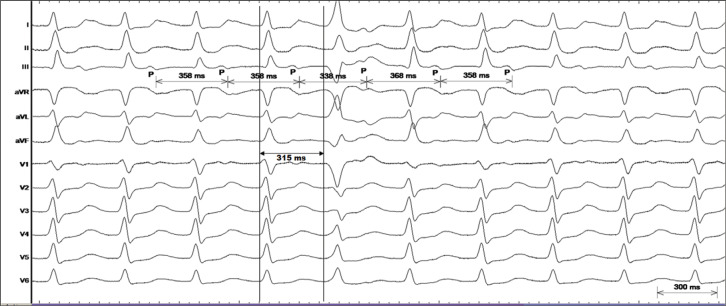
Surface electrocardiogram (I, II, III, aVR, aVL, aVF, V1, V2, V3, V4, V5, V6) during the narrow QRS tachycardia with annotations.

**Figure 3:**
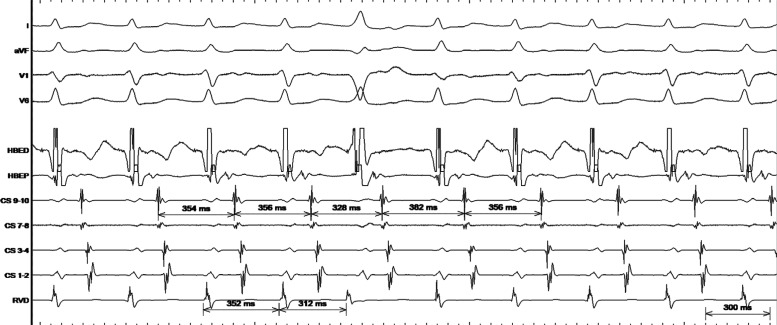
Surface electrocardiogram (I, aVF, V1, V6) and intracardiac electrograms—His bundle electrogram distal (HBED), His bundle electrogram proximal (HBEP), coronary sinus (CS) 9,10 dipoles at CS ostium, CS 1,2 dipoles at distal CS, and right ventricular apex distal (RVD) during the tachycardia.

## References

[1] Mohanan Nair KK, Namboodiri N, Thajudeen A, Valaparambil A, Tharakan J (2016). Narrow QRS tachycardia with long RP interval: what is the mechanism?. Pacing Clin Electrophysiol.

[2] Miles WM, Yee R, Klein GJ, Zipes DP, Prystowsky EN (1986). The preexcitation index: an aid in determining the mechanism of supraventricular tachycardia and localizing accessory pathways. Circulation.

